# Labeling of DOTA-conjugated HPMA-based polymers with trivalent metallic radionuclides for molecular imaging

**DOI:** 10.1186/s13550-018-0372-x

**Published:** 2018-02-27

**Authors:** Elisabeth Eppard, Ana de la Fuente, Nicole Mohr, Mareli Allmeroth, Rudolf Zentel, Matthias Miederer, Stefanie Pektor, Frank Rösch

**Affiliations:** 1 Clinic for Nuclear Medicine, University Medical Center Bonn, Bonn, Germany; 2 Institute of Nuclear Chemistry, Johannes Gutenberg University Mainz, Mainz, Germany; 3 Institute of Organic Chemistry, Johannes Gutenberg University Mainz, Mainz, Germany; 4 Clinic for Nuclear Medicine, University Medical Center Mainz, Langenbeckstraße 1, 55131 Mainz, Germany

**Keywords:** DOTA-HPMA conjugates, Theranostic, Radiolabeling, Gallium-68, Scandium-44, Lutetium-177, PET, Biodistribution

## Abstract

**Background:**

In this work, the in vitro and in vivo stabilities and the pharmacology of HPMA-made homopolymers were studied by means of radiometal-labeled derivatives. Aiming to identify the fewer amount and the optimal DOTA-linker structure that provides quantitative labeling yields, diverse DOTA-linker systems were conjugated in different amounts to HPMA homopolymers to coordinate trivalent radiometals Me(III)* = gallium-68, scandium-44, and lutetium-177.

**Results:**

Short linkers and as low as 1.6% DOTA were enough to obtain labeling yields > 90%. Alkoxy linkers generally exhibited lower labeling yields than alkane analogues despite of similar chain length and DOTA incorporation rate. High stability of the radiolabel in all examined solutions was observed for all conjugates. Labeling with scandium-44 allowed for in vivo PET imaging and ex vivo measurements of organ distribution for up to 24 h.

**Conclusions:**

This study confirms the principle applicability of DOTA-HPMA conjugates for labeling with different trivalent metallic radionuclides allowing for diagnosis and therapy.

**Electronic supplementary material:**

The online version of this article (10.1186/s13550-018-0372-x) contains supplementary material, which is available to authorized users.

## Background

One of the major problems in current chemotherapy is the lack of selectivity of the utilized anticancer drugs. Conventional chemotherapeutic drugs suffer from a narrow therapeutic index as a consequence of affecting not only cancer cells but basically all cells with high proliferation rates. The therapeutic index of a drug can be improved by increasing the accumulation at the target site, e.g., the tumor [1, 2]. Nanomaterials (< 100 nm) can often improve pharmacokinetics due to their particle size, charge, and shape and may passively and/or actively accumulate in tumor tissue. Since the early 1970s, *N*-(2-hydroxypropyl)methacrylamide (HPMA) is investigated as drug delivery system based on the enhanced permeability and retention (EPR) effect [3–5]. HPMA-based copolymers are hydrophilic, biocompatible, and non-immunogenic [6, 7], and selection as drug carrier is based on detailed studies of the relationship between structure and biocompatibility of hydrophilic polymers [8–13]. HPMA-copolymer conjugates have been successfully utilized in the delivery of miscellaneous drugs (e.g., doxorubicin [14], paclitaxel [15], camptothecin [16]). Modification can be achieved through activated comonomers leading to biocompatible polymers with functionalities for polymer analogue reaction. This strategy is very flexible in the choice of ligands, type of activation, and occupancy rate and was already used in various studies [17].

As it was shown before, nanomaterials like HPMA-based nanocarriers are able to combine both imaging agents and drugs within one formulation [8, 18, 19] for the in vivo analysis by positron emission tomography (PET) [20, 21]. Those studies include information about the early pharmacology of fluorine-18-radiolabelled HPMA derivatives, yielding information of excretion pathways, blood retention, and organ distribution. According to the physical half-life of fluorine-18 of 110 min, those data only cover a period of 2 to 4 h post injection. For an investigation of later time points, in particular when drug accumulation in tumors is of interest, radionuclides of longer physical half-life are needed. Here, we intent to introduce a radiolabeling concept based on a chelator being (i) adequate to the coordination chemistry of trivalent metals, namely DOTA (1,4,7,10-tetraazacyclododecane-1,4,7,10-tetraacetic acid), and being (ii) covalently attachable to the HPMA structure.

Two generator-produced positron emitters of choice are gallium-68 and scandium-44, which can be applied to quantitative in vivo imaging via PET. Gallium-68 is a positron emitter (89%) with a half-life of 67.71 min and average positron energy of 1.899 MeV. Its physical half-life is adequate to allow the preparation and purification of gallium-68-labeled radiopharmaceuticals and for imaging biological processes with short half-life. The major advantage of gallium-68 is its availability via a ^68^Ge/^68^Ga-generator system, which provides a cyclotron-independent and cost-effective source of the isotope in suitable conditions for labeling after its post-processing [22–27]. Scandium-44 (94% positron emission) is available via the ^44^Ti/^44^Sc-generator system [28, 29]. Due to its longer physical half-life of 3.97 h, scandium-44 facilitates the tracking of processes with longer biological half-life than gallium-68 or even fluorine-18. It could be applied for more exact planning and dosimetry calculations in endoradiotherapy [27]. In addition, longer-lived lutetium-177 (6.7 days) can be used as well for ex vivo organ distribution studies. Lutetium-177 is not suitable for PET imaging, but as a result of emitting gamma radiation (113.0 and 208.4 keV) in addition to its β^−^ particles, it can be used for therapy and SPECT imaging [30]. Those radiolabeled HPMA derivatives appear also to be suitable to investigate processes such as the EPR-mediated accumulation of nanoparticles in tumor tissue.

In addition, HPMA polymers could also be labeled with iodine-123 [31] to visualize tumors and metastases by SPECT or 99m-technetium [32] as it was done before. To our knowledge so far, no studies exist with iodine-124-labeled HPMA polymers but this radionuclide was already used to enable long-term tumor PET imaging of clinically approved silica nanoparticles [33] and could also be a useful tool for imaging long-circulating HPMA polymers.

In the present study, we aimed to synthesize DOTA-conjugated HPMA homopolymers with alkyl- and alkoxy linkers for labeling with trivalent metallic radionuclides. The main objective was to show how the linker (in terms of structure and length) as well as the amount of incorporated DOTA may influence labeling kinetics, stability, and whether labeling procedures for different radionuclides can be utilized. Labeling of the synthesized DOTA-conjugated homopolymers was first performed with gallium-68 to evaluate reaction parameters (incubation time, amount of compound, temperature, influence of organic solvent). The stability of the gallium-68-labeled conjugates was analyzed in vitro in 0.9% NaCl and human serum albumin (HSA), as well as in the presence of different metal cations and other competing chelators, like EDTA and DTPA. Additionally, labeling with the diagnostic and therapeutic isotopes scandium-44 and lutetium-177 was performed to allow for the characterization of late organ distribution profiles of the HPMA-based nanoparticles in following studies. Proof of principle in vivo distribution was investigated by PET with a HPMA-DOTA conjugate of 13 kDa, a short ethyl linker, and a low DOTA/HPMA ratio (1.6%), which was radiolabeled with scandium-44.

## Methods

All chemicals were analytical or pure reagent grade and used as received unless otherwise specified. DOTA-(^t^Bu)_3_ was obtained from CheMatech (Dijon, France). All other organic and inorganic reagents were purchased from Sigma-Aldrich (Munich, Germany) and Across Organics (Geel, Belgium). Deionized Milli-Q water (18.2 MΩ cm; Millipore, Darmstadt, Germany) was used in all organic reactions. Dioxane was distilled over a sodium/potassium composition. Lauryl methacrylate was distilled to remove the stabilizer and stored at − 18 °C. 2,2′-Azo-bis-(isobutyronitrile) (AIBN) was recrystallized from diethyl ether and stored at − 18 °C as well. ^1^H-NMR spectra were obtained by a Bruker AC 300 spectrometer (Bruker, Karlsruhe, Germany) at 300 MHz. The amount of incorporated DOTA derivatives **17**–**21** in the polymers was determined by inverse gated ^13^C-NMR. ^19^F-NMR analysis was carried out with a Bruker DRX-400 at 400 MHz. It proved the complete conversion of the reactive ester units by reaction with the DOTA derivatives and 2-hydroxy-propylamine in analogy to [34]. The synthesized polymers were dried at 40 °C under vacuum overnight, followed by gel permeation chromatography (GPC). GPC was performed in tetrahydrofuran (THF) as solvent, using the following equipment: pump PU 1580, autosampler AS 1555, UV detector UV 1575, and RI detector RI 1530 from Jasco (Pfungstadt, Germany) as well as a miniDAWN Tristar light scattering detector from Wyatt (Dernbach, Germany). Columns were used from MZ Analysentechnik, 300 × 8.0 mm: MZ-Gel SDplus 106 Å 5 μm, MZ-Gel SDplus 104 Å 5 μm, and MZ-Gel SDplus 102 Å 5 μm. GPC data were evaluated by using the software PSS WinGPC Unity (Polymer Standard Service Mainz, Germany). The flow rate was set to 1 mL/min with a temperature of 25 °C. Commercial ^68^Ge/^68^Ga generators based on TiO_2_ phase absorbing ^68^Ge(IV) were obtained from Cyclotron Co., Ltd. (Obninsk, Russia). Typically, batch activities of 80–150 MBq were used. Scandium-44 was provided from a ^44^Ti/^44^Sc-generator system developed in Mainz [28]. Batch activities of 165–180 MBq were used. Lutetium-177 was obtained from ITG (Munich, Germany) and used without further purification. Counting was performed in a borehole counter (Nuklear-Medizintechnik Dresden GmbH, Germany). Thin-layer chromatography (TLC) was performed on silica-gel (silica-gel 60F254; MERCK, Darmstadt, Germany)-coated aluminum TLC sheets and analyzed using an instant imager (Instant Imager, Canberra Packard, Schwadorf, Austria). The cation exchange resins AG 50W-X8 (− 400 mesh), AG 50W-X4 (200–400 mesh), and AG 50 W–X8 (200–400 mesh) were obtained from Bio-Rad (Munich, Germany). Strata-X mini-C-18 cartridges were obtained from Phenomenex (Aschaffenburg, Germany). HiTrap™ Desalting Columns were purchased from GE-Healthcare Europe GmbH (Freiburg, Germany). TraceSelect water (Sigma-Aldrich, Germany) was used for all aqueous radiolabeling solutions.

### Synthesis and characterization of DOTA-HPMA conjugates

**Fig. 1:**
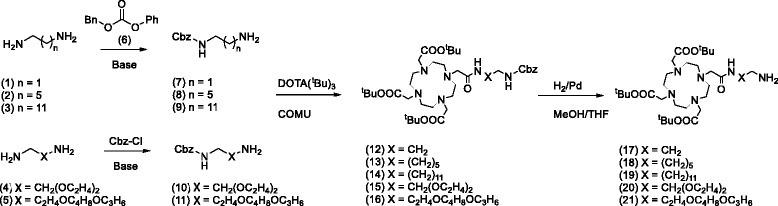
Synthesis of the (^t^Bu)_3_-DOTA linkers (17–21)

**Fig. 2:**
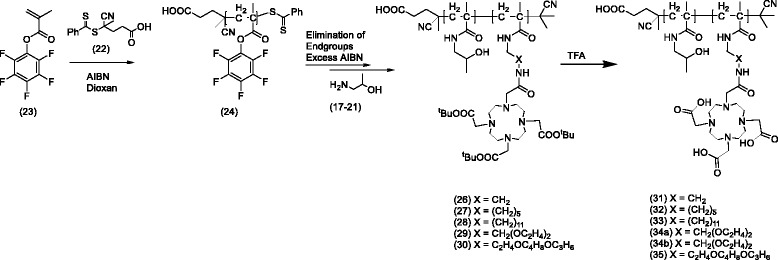
Synthesis of the HPMA-linker-DOTA systems (31–35)

The DOTA-HPMA conjugates were prepared following the synthesis routes depicted in Figs. 1 and 2. Figure 1 shows the preparation of different DOTA linkers that later on were conjugated to the HPMA backbones in different amounts. Details on the synthesis route and characterization are added in the supplementary information. The hydrodynamic radius (Rh) of the polymers (0.1 mg/ml in isotonic NaCl solution) was determined by Fluorescence Correlation Spectroscopy (FCS) at RT.

### Radiochemistry

#### Labeling with gallium-68

Labeling of DOTA-conjugated HPMA homopolymers was performed by mixing aliquots of polymer stock solution (1 mg/mL in water) with post-processed gallium-68 eluate [24, 25]. Investigation of the amount of conjugate needed for labeling reaction was performed by varying time and concentration of DOTA-conjugated polymer using conjugate **31** with acetone post-processed gallium-68 and was adopted for all the other labeling reactions. In addition, labeling with HPMA homopolymer without DOTA functionalization was performed under these conditions to investigate the unspecific binding. Reaction mixtures were heated up to 95 °C for 30 min.

#### Labeling with scandium-44

Labeling of DOTA-conjugated HPMA homopolymers was performed with 250 μL polymer stock solution (1 mg/mL in water) in post-processed scandium-44 eluate (3 mL) and heating up to 95 °C for 30 min [35].

#### Labeling with lutetium-177

Labeling of DOTA-conjugated HPMA homopolymers was performed with 250 μL polymer stock solution (1 mg/mL in water) with 150 μL 0.1 M Na acetate buffer (pH = 8) and 100 μL lutetium-177 and heating up to 95 °C for 30 min. The stoichiometric ratio polymer-to-lutetium-177 was 10:1.

#### Purification of the labeled conjugates

The radiolabeled polymeric systems were cleaned from unreacted radiometal by Sephadex G-25 size exclusion chromatography (HiTrap™ Desalting Column, Sephadex G-25 Superfine, 0.9% NaCl, flow rate: 0.5 mL/min) leading to a pure, gallium-68-labeled polymer solution ready for subsequent experiments.

#### Quality control

Quality control of complex formation was performed by radio thin-layer chromatography (radio-TLC). The TLC plates were developed with 0.1 M citric buffer (pH = 4). Quantitative distribution of radioactivity on TLC plates was measured using an instant imager.

#### Stability of the labeled compounds

Stability studies were performed with gallium-68-labeled polymers of radiochemical purity > 98% in physiological conditions and in the presence of competing metals and chelates. Detailed setup is explained in the supplementary section. As nonspecific binding of the metal cation to nanoparticles is a basic problem in labeling those systems, investigation of nonspecific binding of gallium-68 to a HPMA polymer was performed with 20 nmol HPMA homopolymer without functionalization (Mn: 12.000 g/mol, 0% DOTA) utilizing the optimized protocol.

### In vivo PET imaging and ex vivo biodistribution

Six–8-week-old male C57BL/6J (Janvier, France) mice were housed under specific pathogen-free conditions in the animal care facility in Mainz according to the guidelines of the regional animal care committee. All experiments were performed in accordance with federal guidelines and approved by the ethical committee of the state of Rheinland-Pfalz (according to §8 Abs. 1 Tierschutzgesetz, Landesuntersuchungsamt; permission no. 23177-07/G121-092 to M. Miederer). All injections and imaging experiments were done under isoflurane anesthesia to minimize suffering. Mice were injected subcutaneously with 2 × 10^5^ murine B16-F10 melanoma tumor cells into the right flank. When tumors reached a volume of 150–200 mm^3^, ^44^Sc-labeled polymer conjugate 31c (1.5 mg in 1 mL of isotonic saline) was injected i.v. in anesthetized mice with a mean activity of 4.6 ± 0.5 MBq. Whole body scandium-44 PET scans were dynamically acquired over 120 min post injection (p.i.), or 30-min static scans were performed 90 min, 210 min, and 24 h p.i.

All PET imaging studies were performed on a nanoScan PET/MRI (Mediso, Budapest, Hungary), and PET data were dynamically reconstructed with Teratomo 3D (4 iterations, 6 subsets, voxel size 0.4 mm) using user defined frames (3 × 20; 3 × 60; 3 × 120; 10 × 300; 6 × 600 s) or statically using one 30-min frame. Afterwards, the PET images were analyzed with pmod software (version 3.6).

After 2, 4, and 24 h, the animals were sacrificed and different organs and blood were removed. The tissue samples were weighed and the ^44^Sc activity in the organs was directly measured in a γ-counter (2470 WIZARD2 Automatic Gamma Counter, PerkinElmer) to calculate the percentage of accumulated activity as % ID/g.

### Ex vivo metabolism evaluation

Blood samples (ca. 100 μl), collected during the biodistribution, were mixed with heparin solution (150 μl) to avoid coagulation. Blood samples were weighed and activity was measured in a γ-counter; 500 μl of PBS were added and the blood was centrifuged at 1700×*g* for 3 min in order to separate blood cells and plasma. The plasma fractions were weighed and activity was measured in a γ-counter. Proteins and polymers were precipitated by adding the same volume of acetonitrile to the plasma fraction. To separate proteins and polymer from plasma water, the samples were centrifuged at 1700×*g* for 3 min. The supernatants were weighed followed by determination of the activity in a γ-counter. The percentage of radioactivity in the blood cells, protein plus polymers, and plasma water fractions was calculated by subtracting the activities of the supernatants from the activity of the whole blood sample.

## Results

### Organic synthesis

For coupling reaction between the HPMA backbone and the bifunctional chelator, orthogonal amidation chemistry via active ester groups was selected [17]. Synthesis of the amine-terminated DOTA-based ligands **17**–**21** from commercially available (^t^Bu)_3_-DOTA is depicted in Fig. 1.

First, the commercially available α,ω-diamines **1**–**5** were mono-Cbz-protected. The protection was realized with benzylphenylcarbonate **6** for **1**–**3** (Additional file 1) and with benzylchloroformate for **4**–**5**. The selectivity of the mono-Cbz-protection using **6** decreases with the length of the alkyl chain but achieves good yields for **1**–**3**. Mono-Cbz-protection with **6** was ineffective for the alkoxy diamines **4**–**5** so benzylchloroformate was used instead obtaining good yields of the desired product. The following amidation of (^t^Bu)_3_-DOTA with **7**–**11** afforded the Cbz-protected (^t^Bu)_3_-DOTA ligands **12**–**16** in good yields of 66–79%. The Cbz-protecting group was removed via hydrogenation to obtain the (^t^Bu)_3_-DOTA ligands **17**–**21** in almost quantitative yields (93–97%).

The synthetic route to the DOTA-HPMA conjugates is depicted in Fig. 2. Starting from pentafluorophenyl methacrylate monomer **23**, the precursor homopolymer **24** was synthesized according to literature (Additional file 1). The dithioester end group was removed by an excess of 2,2′-azo-bis-(isobutyronitrile) as described in literature (Additional file 1) taking the advantage of circumventing side reactions during the next reaction steps. Functionalization of the polymeric precursor was obtained by aminolysis via the amine-terminated (^t^Bu)_3_-DOTA ligands **17**–**21**. The incorporation was calculated to be between 0.6–11% DOTA for each conjugate by inverse gated ^13^C-NMR spectroscopy. Addition of 2-hydroxypropylamine leads to the polymeric structures **26**–**30**. The entire conversion of the reactive ester groups was proved by means of ^19^F-NMR spectroscopy, which demonstrates complete disappearance of ^19^F signals of the polymeric backbone in analogy to [34]. As these reactions were performed in water-free conditions, they happen without side reactions [34]. In a final step, the chelator was deprotected using TFA. The pure HPMA-DOTA conjugates, ready for labeling, could be obtained as white powders with good yields of 89%, except conjugate **32**, which could not be recovered from the dialysis flexible tube.

As intended, five different DOTA-conjugated polymer conjugates with relatively low polydispersity index (PDI) and narrow molecular weight distribution (*R*_*h*_: 1.3–1.5 nm) have been achieved by RAFT polymerization and successfully functionalized in a following polymer analogous reaction. Their molecular weight average numbers (Mn), polydispersity indices (PDI), and % of incorporated chelators are shown in Table 1.

**Table 1:** Analytical data of the final HPMA-DOTA conjugates 31–35

Polymer	Mn [g/mol]	PDI^a^	% DOTA^b^	Linker
31	15.000	1.3	11	(1)
33	12.000	1.2	1.6	(3)
34a	12.000	1.2	0.6	(4)
34b	12.000	1.2	1.2	(4)
35	13.000	1.2	3.5	(5)

^a^Determined by GPC in THF as solvent

^b^Determined by ^1^H-NMR spectroscopy after polymer analogous reaction with 2-hydroxypropylamine

Later on, in order to have only one variable for direct comparison (the conjugates so far presented different linker structures with different incorporation rates of DOTA), conjugate **31** was modified with different percentages of DOTA, affording the conjugates **31a**–**31c** following the same synthesis route as described for conjugate **31** (Table 2).

**Table 2:** Analytical data of the final HPMA-DOTA conjugates (derivatives of type 31)

Polymer	Mn [g/mol]	PDI^a^	% DOTA^b^	Linker
31	15.000	1.2	11	(1)
31a	14.000	1.2	4.8	(1)
31b	13.000	1.2	3.3	(1)
31c	13.000	1.2	1.6	(1)

^a^Determined by GPC in THF as solvent

^b^Determined by ^1^H-NMR spectroscopy after polymer analogous reaction with 2-hydroxypropylamine

### Radiochemistry

**Fig. 3:**
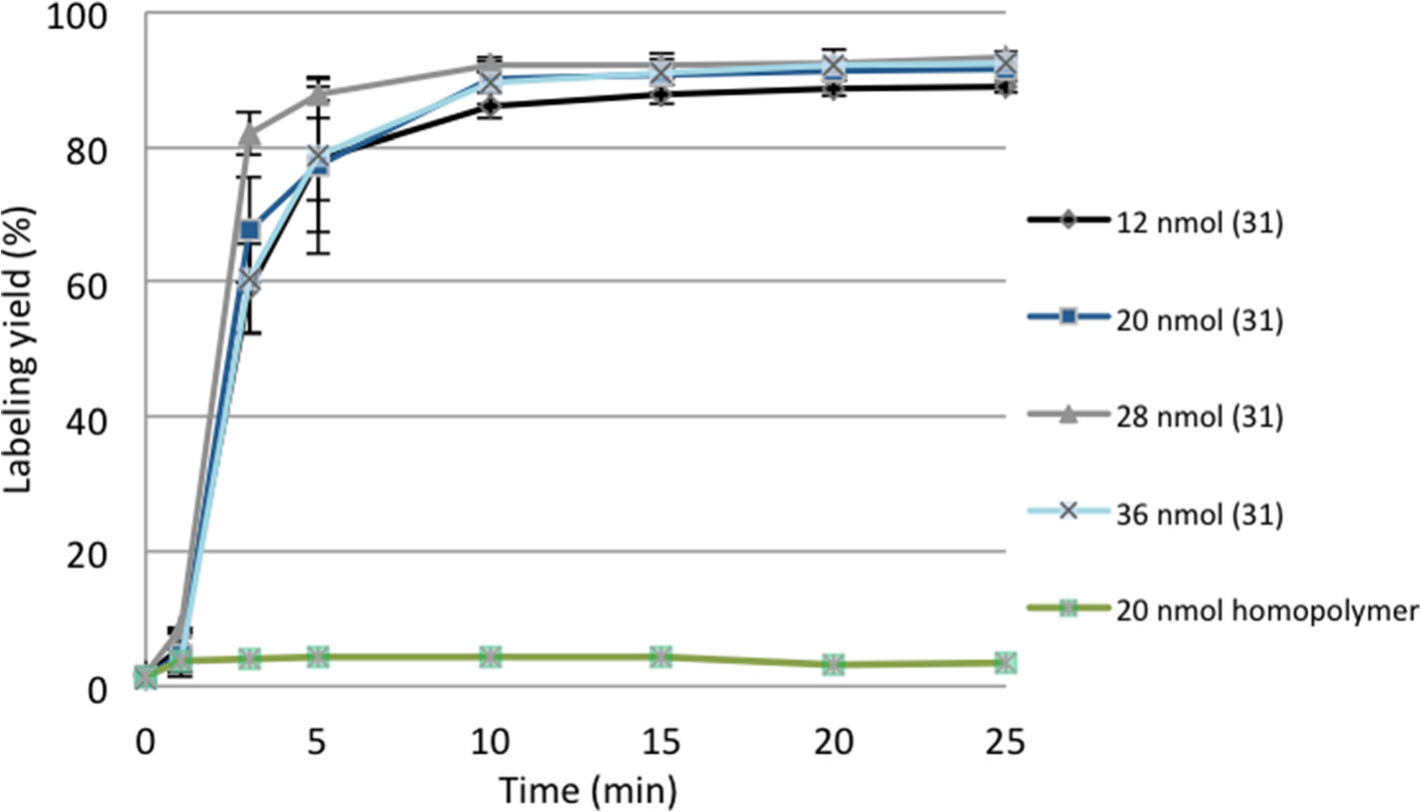
Time course of ^68^Ga-complex formation with varying amounts of DOTA-HPMA conjugate 31 and polymer (20 nmol) not modified with DOTA as a control

DOTA derivatives generally require elevated temperatures for complex formation in contrast to acyclic chelators; 95 °C was used as standard temperature for labeling experiments with the DOTA-HPMA conjugates with different radionuclides (e.g., gallium-68, scandium-44, lutetium-177). Figure 3 shows the kinetics of [^68^Ga]Ga-**31** complexation depending on the amount of the DOTA-HPMA **31** ranging from 12 to 36 nmol. The accessible labeling yield is not influenced by the amount of conjugate used. Only 12 nmol showed a slightly lower labeling yield. Thereon, in order to work with the fewest amount of polymer while achieving maximal labeling yield in a short time, 20 nmol was used for further experiments. Labeling with generator-derived and acetone post-processed gallium-68 in water is accomplished within 15 min at 95 °C with RCY of 90%. The complex formation is fast and efficient. Investigation of nonspecific binding of gallium-68 to a HPMA polymer was performed with 20 nmol HPMA homopolymer without DOTA functionalization. Within 25 min, less than 5% of the radioactivity binds to the HPMA homopolymer (Fig. 3).

**Fig. 4:**
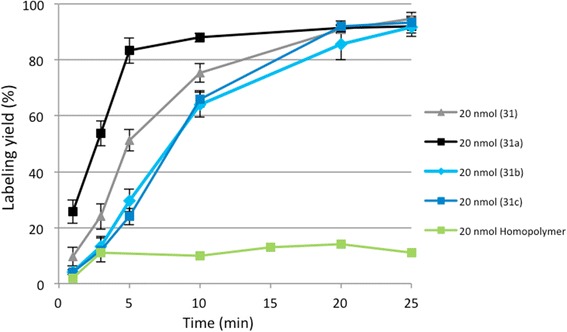
Radiolabeling yield of conjugates 31 with different amounts of DOTA utilizing acetone post-processed gallium-68 (20 nmol; 25 min; 95 °C; *n* = 3)

Next, it was tested if the radiolabeling yields are influenced by the DOTA incorporation rate in polymers with the same linker length (Fig. 4). It can be observed that radiolabeling kinetics is not directly proportional to the amount of incorporated chelator. The fastest radiolabeling kinetics are shown for conjugate **31a** with 4.8% DOTA. Slower kinetics are achieved for conjugate **31** with 11% DOTA and slightly slower radiolabeling kinetics for the polymer with 1.6% and with 3.3% of incorporated DOTA. After 25 min, all conjugates reach the same labeling yield and do not show further differences.

**Fig. 5:**
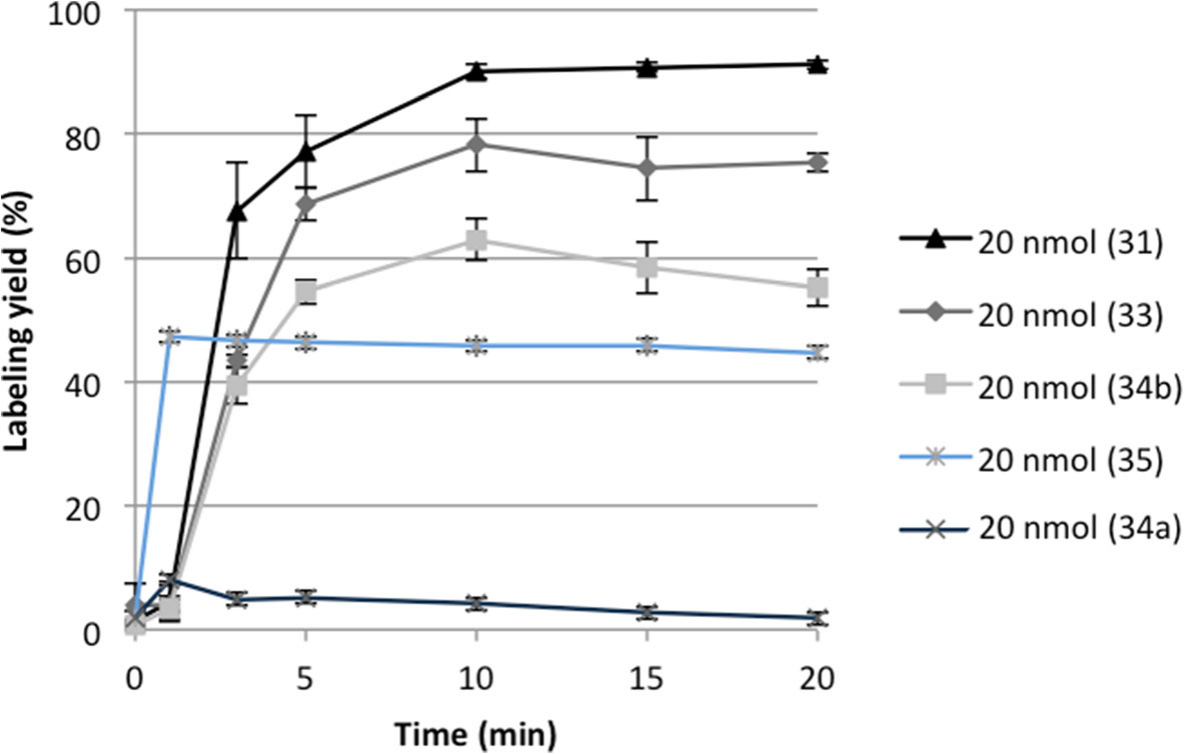
Labeling yield using acetone post-processed gallium-68 (20 nmol; 15 min; 95 °C; *n* = 3)

One could hypothesize that labeling efficiency will be related to linker length and linker structure. To test this, DOTA-HPMA conjugates **31**, **33**, **34a/b**, and **35** were labeled using the same conditions as before. Within 15 min, labeling yields of 44–92% can be achieved (Fig. 5, Table 3). The highest labelling yields can be found for conjugate **31** with the shortest alkyl linker (91%) and highest DOTA content (11%) followed by conjugate **33** with the longest alkyl linker (75%) and 10-fold lower DOTA content (1.6%). This shows that labeling yield is affected by the length of the linker as well as the DOTA content. Furthermore, all conjugates with alkoxy linkers showed distinct lower radiolabeling yields compared to those with alkyl linkers whereupon conjugate **34b** shows slightly higher labelling yield than conjugate **35** with the longer alkoxy linker showing again the influence of the linker length. Conjugate **34a** with a very low DOTA content (0.6%) shows almost no labeling.

**Table 3:** Labeling of HPMA-DOTA conjugates 31–35 using acetone and ethanol post-processed ^68^Ga eluate (20 nmol, 20 min, 95 °C)

Polymer	Linker	% DOTA	RCY acetone PP (%)	RCY ethanol PP (%)
31	(1)	11	91 ± 1	98 ± 1
33	(3)	1.6	75 ± 2	97 ± 1
34a	(4)	0.6	2 ± 1	25 ± 8
34b	(4)	1.2	55 ± 3	40 ± 8
35	(5)	3.5	45 ± 22	67 ± 6

Recently, the benefit of additional use of organic solvents to facilitate radiolabeling was proven [36] and utilized in an ethanol-based post-processing. It has been shown that this ethanol-based post-processing protocol significantly increases radiochemical yields even at lower temperature compared to the standard procedure for DOTATOC as model compound [26]. To take the advantage of additional ethanol, enhancing labeling yields, conjugates **33**–**35** were labeled utilizing this protocol too. Additional file 1: Figure S1 shows the results of labeling conjugate **33** utilizing the two post-processing methods. Labeling with the ethanol post-processed gallium-68 in 1 M NaHEPES buffer leads to significantly increased yields of complex formation. The difference between both labeling procedures is accounted for 20%. An additional advantage of the ethanol-based post-processing is the possibility to reduce the overall volume of the labeling solution from 5.25 mL (acetone) to 2.25 mL (ethanol), and therefore to increase the specific activities.

This shows that labeling is also affected by the linker structure. Conjugates **33** and **35** have the same chain length (*n* = 11) but differ in their structure. Conjugate **33** contains a dodecyl linker while the positions 4 and 9 are substituted with oxygen atoms in the linker chain of **35**. This structural difference is reflected in the decreasing labeling yield from 75% **33** to 45% **35**.

#### Purification of gallium-68-labeled DOTA-HPMA conjugates and quality control

After labeling, the polymeric systems could be purified fast and easy using Sephadex G-25 size exclusion chromatography. Over 90% of the radiolabeled product can be collected in two fractions with radiochemical purity of > 98%. As the product is eluted from the column with 0.9% NaCl, the resulting solution could be directly used for in vivo studies. Radiochemical analysis of complex formation was performed by TLC. The *R*_*f*_ values for free uncomplexed gallium-68 and labeled conjugates were determined as *R*_*f*_ ([^68^Ga]Ga^3+^) = 0.9 and *R*_*f*_ (^68^Ga conjugate) = 0. The *R*_*f*_ values for scandium-44 and lutetium-177 utilizing the same method are the same as for gallium-68, 0.9.

#### Radiolabeling with scandium-44

**Fig. 6:**
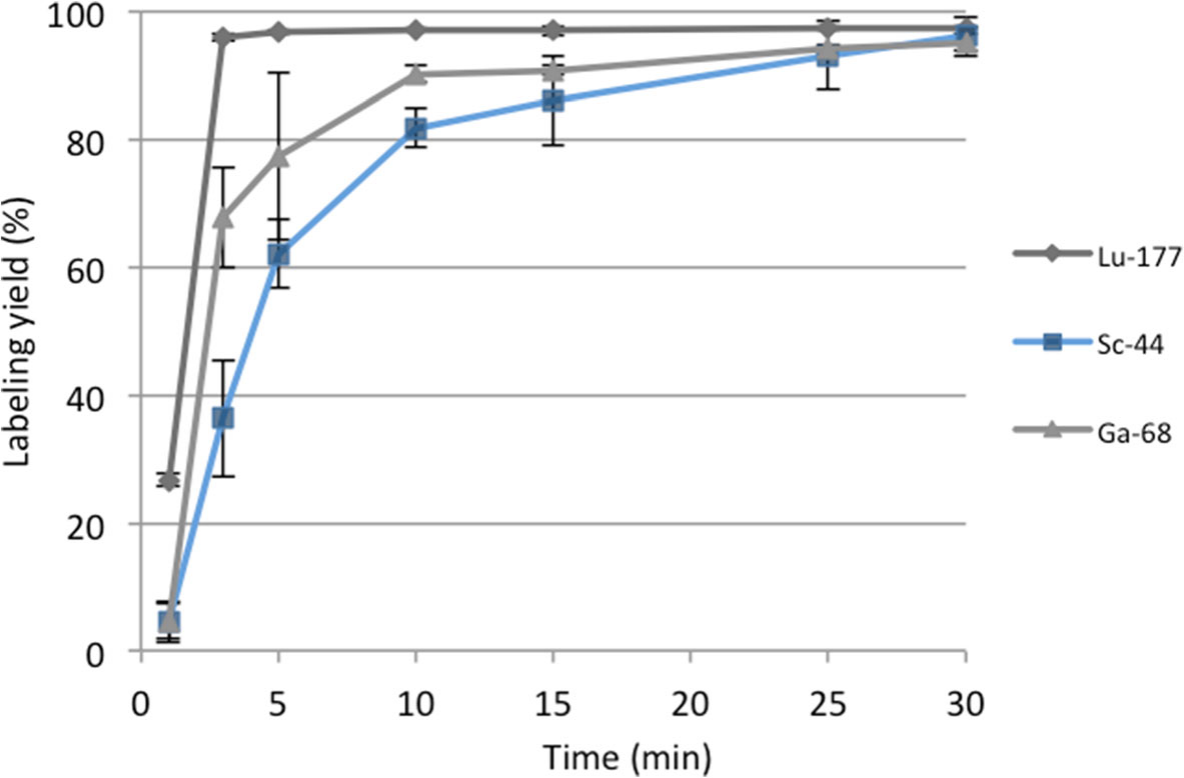
Labeling yields for 31 with scandium-44, gallium-68, and lutetium-177 (20 nmol; 30 min; 95 °C; *n* = 3)

As conjugate **31** showed the best radiolabeling yield, this conjugate was used for further labeling experiments with scandium-44 and lutetium-177. Scandium belongs to the transition metal group, and its complex formation strongly depends on pH of aqueous solution as previously shown for [^44^Sc]Sc-DOTATOC complex formation [35]. Utilizing the post-processing method leads to a final solution of scandium-44 in ammonium acetate buffer (pH 4) suitable for direct labelling [29]. Labeling with post-processed scandium-44 in ammonium acetate buffer (pH 4) yields up to 95% within 30 min for DOTA-HPMA **31** (Fig. 6).

#### Radiolabeling with lutetium-177

Radiolabeling with lutetium-177 is fast and efficient. Within 3 min, reaction is completed and yields up to 97% can be obtained for DOTA-conjugated HPMA **31** (Fig. 6).

### Stability of labeled compounds

Stability of the formed complex is a crucial factor in the development of new radiopharmaceuticals. Therefore, several experiments were done to explore stability of the labeled conjugates in NaCl (used as final solvent), human serum albumin, against trans-metalation (Fe^3+^; Ca^2+^; Mg^2+^) and trans-chelation (EDTA, DTPA).

**Fig. 7:**
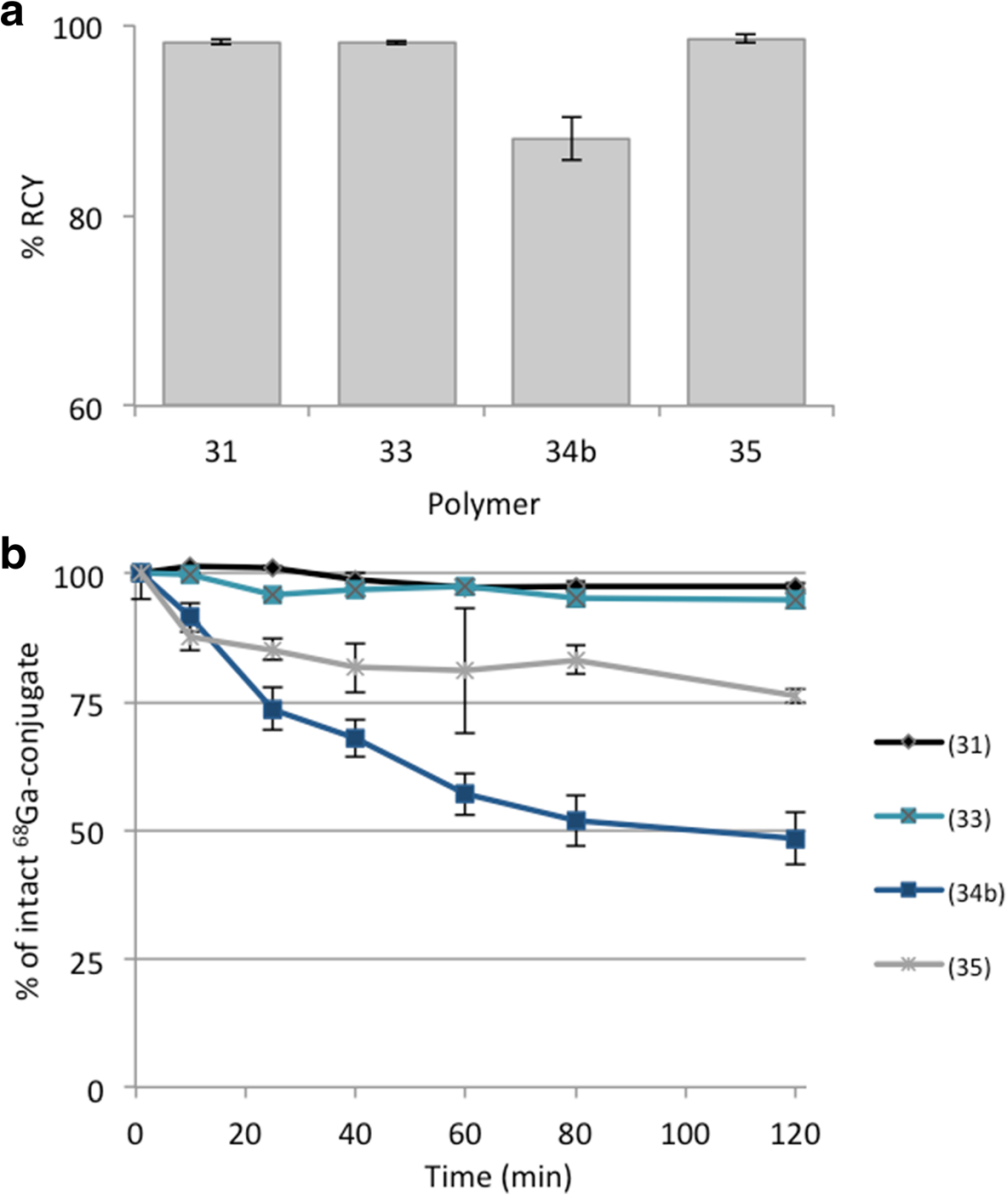
Stability of gallium-68-labeled conjugates in 0.9% NaCl (**a**) and in HSA (**b**) within 120 min at 37 °C (*n* = 3)

Stability of gallium-68-labeled DOTA-HPMA conjugates in 0.9% NaCl is presented in Fig. 7a. It indicates a high stability of the labeled conjugates even after 2-h incubation at 37 °C.

In human serum albumin, the conjugates with alkyl linkers **31** and **33** indicate high stability. For both conjugates, the percentage of intact gallium-68 conjugate is higher than 95%. Contrary to that, the conjugates with alkoxy linker structure show higher decomposition rate (Fig. 7b).

The presence of different metal cations (e.g., Fe^3+^) in the final solution or in the blood can cause trans-metalation of the radiolabeled conjugate and finally lead to a release of free radionuclide into solution. Therefore, it is necessary to determine whether the conjugate forms stable complexes in the presence of relevant metal cations or not prior to in vivo studies.

Conjugate **31c** shows the highest stability against trans-metalation (Table 4). Similar to studies of **34**–**35** in HSA, stabilities are affected by addition of other metal cations. Within 120 min, decomposition up to 30% can be observed. More complex are the results for **33**. This conjugate indicates high stability in experiments with HSA, Fe^3+^, and Ca^2+^ while decomposition is observed for challenge studies against Mg^2+^.

**Table 4:** Stability of gallium-68 conjugates at 37 °C in the presence of different metal cations at 10^− 2^ M concentrations (*n* = 3)

Conjugate	Linker	% DOTA	% of intact ^68^Ga conjugate ± SD		
			Fe^3+^	Mg^2+^	Ca^2+^
31c	(1)	1.6	94 ± 3	92 ± 1	92 ± 5
33	(3)	1.6	94 ± 9	69 ± 2	99 ± 2
34a	(4)	0.6	88 ± 2	80 ± 3	70 ± 1
34b	(4)	1.2	84 ± 2	86 ± 2	90 ± 1
35	(5)	3.5	74 ± 2	85 ± 1	83 ± 8

Similarly, stability against other complexing agents like EDTA and DTPA was investigated to check if trans-metalation from HPMA-DOTA conjugate to competing ligand occurs. The results are shown in Table 5. Here, conjugates **34a** and **34b** show trans-metalation to DTPA with more than 30%. All other conjugates are relatively stable to both agents.

**Table 5:** Stability of ^68^Ga conjugates at 37 °C in the presence of EDTA or DTPA at molar ratio 100:1 of competing ligand to conjugate (*n* = 3) after 120 min

Conjugate	Linker	% DOTA	% of intact ^68^Ga conjugate ± SD	
			EDTA	DTPA
31c	(1)	1.6	92 ± 5	83 ± 1
33	(3)	1.6	90 ± 2	87 ± 1
34a	(4)	0.6	77 ± 2	59 ± 1
34b	(4)	1.2	80 ± 3	61 ± 1
35	(5)	3.5	78 ± 5	93 ± 1

Furthermore, one polymer (31c) was labeled with scandium-44 as described in Section 3.2.2, and its stability was evaluated in 0.9% NaCl and HSA for up to 24 h at 37 °C in order to use this polymer for downstream in vivo applications. Table 6 shows that the ^44^Sc-labeled polymer is very stable over a time period of up to 24 h with values > 94% in NaCl and HSA.

**Table 6:** Stability of ^44^Sc conjugate 31c at 37 °C in 0.9% NaCl and HSA during 24 h (*n* = 3)

Time (h)	% of intact ^44^Sc conjugate ± SD	
	0.9% NaCl	HSA
1	98.7 ± 0.5	96.7 ± 0.9
4	98.3 ± 0.9	95.7 ± 0.5
24	97.7 ± 0.5	94.3 ± 0.5

### In vivo and ex vivo biodistribution

**Fig. 8:**
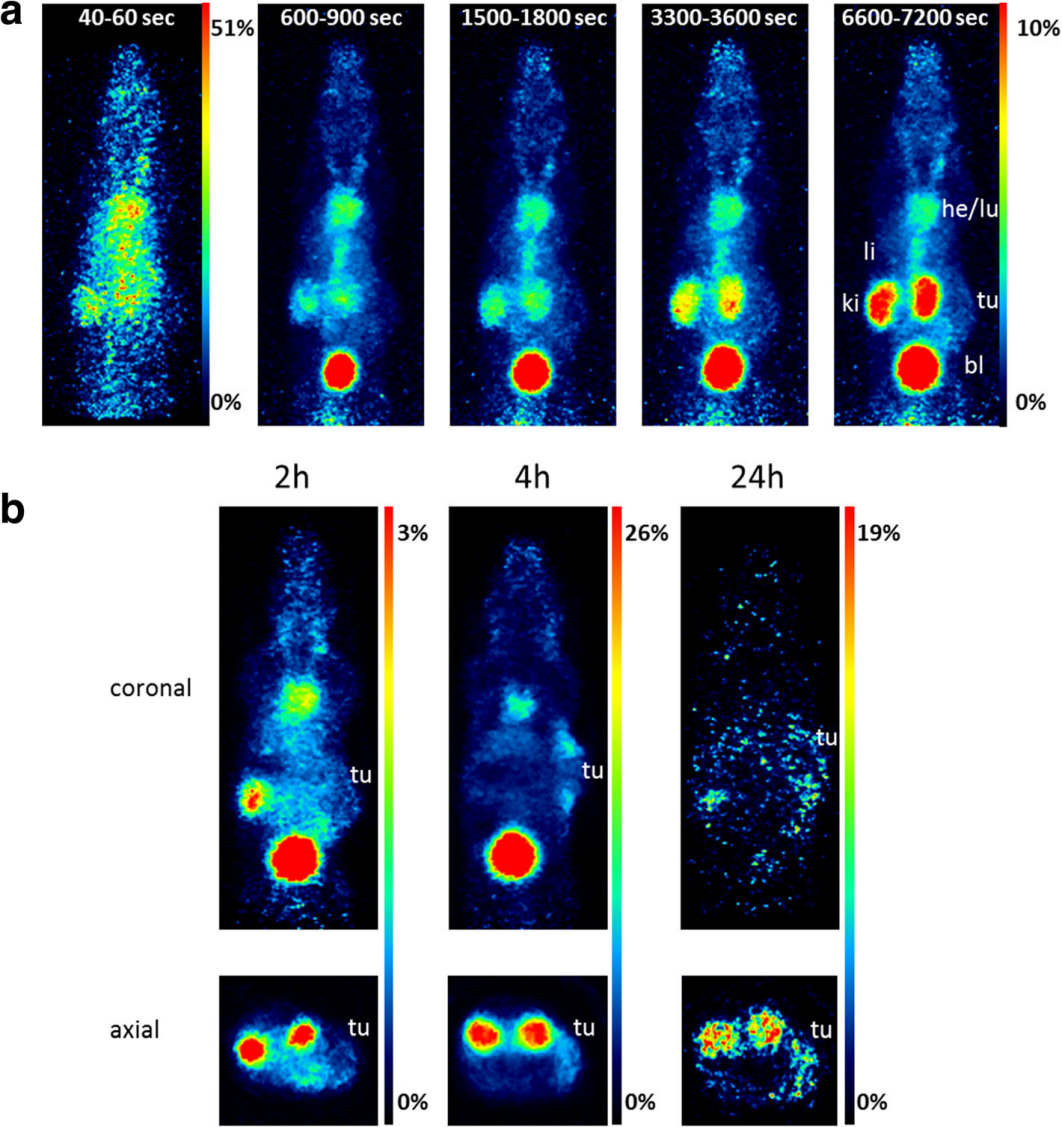
**a** In vivo dynamic PET of [^44^Sc]Sc-DOTA-HPMA 31c in a tumor-bearing mouse (*n* = 1). Maximum intensity projections (MIPs) of different time points during 2-h acquisition. **b** Representative static PET images of [^44^Sc]Sc-DOTA-HPMA 31c in tumor-bearing mice 2, 4, and 24 h p.i. (the last 30 min is imaged). Coronal and axial slices are shown with focus on tumor

For the biological evaluation, ^44^Sc-labeled derivatives were considered in order to demonstrate that the use of trivalent radiometals is favorable in extending the time period of molecular PET imaging compared to ^18^F-labeled analogues without changing the pharmacokinetics. Because of the minimal alterations to structure and geometry and very good radiochemical purity and stability (Table 6), the ^44^Sc-labeled DOTA-HPMA polymer **31c** with the lowest DOTA incorporation and a short alkyl linker was chosen and evaluated in B16 tumor-bearing mice. The dynamic PET acquisition over a time course of 2 h p.i. shows a fast renal excretion of the tracer (Fig. 8a) due to the low molecular weight of the HPMA homopolymer. The heart still shows a robust signal 2 h p.i. while the tumor hardly gets visible over time.

The pharmacokinetics over 24 h shows a relatively low but constant tumor uptake over 4 h and is still visible after 24 h (Fig. 8b) while the uptake in heart declines. The coronal slices show that the tracer mainly accumulates in the outer rim of the tumor after 4 and 24 h. This hints at a poor vascularization inside the tumor what could also be observed during the dissection of the tumors.

**Fig. 9:**
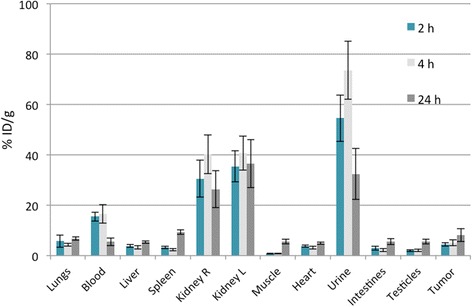
Ex vivo biodistribution of [^44^Sc]Sc-DOTA-HPMA 31c in tumor-bearing mice (*n* = 5 for each time point)

These results are supported by the ex vivo evaluation of the different tissues (Fig. 9, Additional file 1: Table S1).

A fast clearance through the kidneys is observed after 2 h with values of 32.9 ± 6.6% ID/g (urine: 54.6 ± 9.2% ID/g) staying constantly over time. After 24 h, the activity in urine dropped to 32.4 ± 10.2% ID/g.

Uptake in the lungs, liver, and heart remained considerably constant during the three time points studied with mean values of 5.6 ± 1.2, 4.2 ± 0.5, and 4.0 ± 0.5% ID/g (mean values for each organ over time) respectively. Uptake in the spleen increased after 24 h when compared to the earlier time points. The tumor uptake after 2 h p.i. was of 4.4 ± 0.8% ID/g and slightly increased to 5.1 ± 1.7% ID/g after 4 h p.i. The blood values are comparable between 2 h (15.5 ± 1.8% ID/g) and 4 h p.i (16.6 ± 3.7% ID/g). Interestingly, the tumor uptake further increases after 24 h (to 8.2 ± 3.1% ID/g) while tracer concentration in the blood decreases (5.5 ± 1.5% ID/g).

Taken together, ^44^Sc-DOTA labeling of HPMA homopolymers is well suited for measuring the body distribution for a time period of up to 24 h in vivo and ex vivo.

### Ex vivo metabolism

**Fig. 10:**
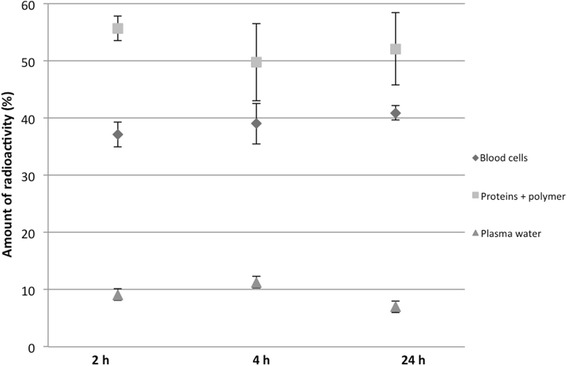
Distribution of [^44^Sc]Sc-31c in the blood for up to 24 h (*n* = 3). Data is expressed as percentage of the amount of radioactivity that was present in the whole blood sample (mean ± SEM)

In order to investigate the in vivo stability of the labeled polymer with scandium-44, the blood samples obtained during biodistribution were separated into different fractions: blood cells, polymer + proteins, and plasma water. Figure 10 shows the amount of radioactivity in the different fractions with reference to the total amount of radioactivity in the blood samples of ^44^Sc conjugate **31c**.

The fraction containing proteins and protein-bound polymer presents 55.2 ± 1.4% of total activity in blood 2 h p.i. This value remained stable after 4 and 24 h p.i. with values of 49.8 ± 6.6% and 52.1 ± 6.3% respectively. In the blood cell fractions, the activity also remained constant during the three time points analyzed (values ranged from 37.2 ± 2.2% to 40.1 ± 1.3%). Only a small proportion of about 10% was found in the plasma water comprising of free scandium-44 and maybe a small amount of [^44^Sc]Sc-HPMA that could not be precipitated completely indicating a good in vivo stability.

## Discussion

Optimized conjugation of DOTA onto HPMA backbone yielded several DOTA-linker-poly(HPMA) conjugates with different linker structures and with variation of amount of DOTA. These derivatives were labeled with various metallic radionuclides (gallium-68, scandium-44, lutetium-177). Labeling and stability of the produced conjugates were investigated in detail with the generator-derived PET radionuclide gallium-68.

It was observed that labeling kinetics with gallium-68 slightly depend on the incorporation rate of DOTA. There seems to be a kind of an optimum amount of 4.8% DOTA for a very fast labeling and at the same time 11% DOTA is too much to efficiently coordinate gallium into the chelator and 1.6% DOTA is insufficient to quantitatively bind gallium, but after 25 min, no differences between the various DOTA amounts were evident. This shows that a minimum of 1.6% DOTA is enough to obtain labeling yields higher than 90%. The linker structure also is a variable that influences labeling efficiency. Alkoxy linker structure generally exhibits lower labeling yields than alkane linkers despite of similar chain length and DOTA incorporation. Moreover, it was observed that a short linker (two-carbon chain) is enough to provide quantitative radiolabeling yields. Another investigated effect is the radiolabeling in the presence of ethanol, which is known to improve labeling efficacy. Utilizing the ethanol post-processed ^68^Ga eluate enhances the labeling efficacy for all conjugates. In conclusion, ca. 1.6% of DOTA per HPMA polymer allow achieving quantitative radiolabeling, if the optimal conditions are applied.

With regard to future in vivo evaluations, special efforts were focused on stability studies of ^68^Ga-labeled conjugates, to adopt the successful candidate to scandium-44 (PET imaging) and lutetium-177 (therapy). Only the conjugates with alkyl chains as linker structure provide high stability in all examined solutions: 0.9% NaCl, HSA, metal cations (Fe^3+^, Mg^2+^, Ca^2+^), and competing ligands (EDTA, DTPA). In general, alkoxy linkers show lower labeling yields and stability than alkyl linker conjugates irrespective of DOTA content. Within the group of conjugates containing alkyl linkers, DOTA content seems to have a mild impact on labeling yield because there is a little difference between the conjugate containing 11 and 1.6% DOTA. In the conjugates containing alkoxy linkers, there might be a kind of threshold for DOTA concerning labeling yield. At 0.6%, almost no labeling could be achieved whereas with 1.2%, high labeling yields were evident. Furthermore, the structure of the linker has more influence on labeling yield and stability than the linker length. That can be explained as a possible unstable complexation of Ga(III) with the oxygen present in the linker. Our hypothesis is that the oxygens on the HPMA structure could induce a more aggregated structure with alkoxy linkers compared to those without oxygens, maybe by van der Waals interaction as it was shown for other biopolymers [37], thus hindering the DOTA availability for the metals. Furthermore, there is no competition between the oxygens and the DOTA. More likely, the van der Waals interactions slightly reduce the complexation of DOTA with the metal.

The conjugate **31c** was also labeled with scandium-44 and further analyzed in vivo because of the minimal alterations to structure and geometry and very good radiochemical purity and stability. The polymer shows a fast renal clearance in vivo.

These findings of the in vivo measured tracer kinetics are in line with earlier data on low molecular weight HPMA homopolymers, which were labeled with fluorine-18 and showed comparable pharmacokinetics after 2 h [20]. By labeling with the longer-lived scandium-44, imaging time could be prolonged which favors imaging of slower molecular processes like the accumulation of targeted drugs into tumors or the activation of T cells after a special vaccination.

HPMA-copolymer conjugates have been successfully utilized in the delivery of miscellaneous drugs (e.g., doxorubicin [14], paclitaxel [15], camptothecin [16]). Modification can be achieved through activated co-monomers leading to biocompatible polymers with functionalities for polymer-analogue reaction. This strategy is very flexible in the choice of ligands, type of activation, and occupancy rate and was already used in various studies [17]. Reaction of a reactive ester polymer with several ligands leads to multifunctionalized copolymers which can be applied for theranostic applications. Recently, a synthetic pathway was shown that combines controlled radical polymerization with post-polymerization modification of a perfluorinated reactive ester [38] to get access to well-defined HPMA-based block copolymers of low dispersity and controlled copolymer structure with a high degree of multiple functionalities. With this technology, advanced HPMA-based polymer conjugates and nano-sized self-assemblies could be developed that may serve as novel drug carriers. Moreover, the high degree of functionalities enables novel applications for HPMA-based copolymers, for instance, in the field of tumor immunotherapy [39].

Interestingly, despite the low molecular weight, without any expected EPR effect, tumor accumulation slightly increased over time while tracer concentration in the blood decreased. The enhanced tumor accumulation could be due to the higher permeability reported for B16F10 tumors compared to any other tumor types [40]. However, this 24-h blood value is also well comparable to earlier data by Lammers et al. [41] were they labeled HPMA copolymers of different weights by iodination (iodine-131) and discovered the body distribution up to 168 h p.i. [41]. Here, the smallest evaluated polymer (23 kDa) showed a similar blood retention after 24 h like the HPMA polymer in our study even though those studies were performed in rats and with another tumor model.

Interestingly, our findings on the different blood components suggested that the homopolymer interacts with serum proteins and with blood cells. Former studies of our group showed that HPMA-*ran*-LMA copolymers interact with very low density lipoproteins in the blood [42] and with cell membranes [43, 44]. Not only structure and size of the polymer but furthermore the cell type is crucial for polymer delivery. Moreover, hydrophilic homopolymers (like in our case) were taken up by micropinocytosis as well as by clathrin-mediated and clathrin-independent endocytosis [45].

Furthermore, it is well known that nanoparticles and polymers are opsonized by plasma proteins upon entering the blood stream [46, 47] which alters their molecular size, charge, and aggregation behavior and thereby their physicochemical properties dramatically. One could imagine that the HPMA polymers used in this study get “sticky” by the protein corona and may bind unspecifically to cells in the blood stream (monocytes, granulocytes, B and T cells) or may even be endocytosed. Especially, when polymers are opsonized by complement factors, which were also identified in the protein corona of nanocarriers [48, 49], they will be taken up rapidly by circulating cells of the immune system. Several in vitro studies showed that dendritic cells can bind and take up HPMA-based homopolymers [39, 50] and even other immune cells like granulocytes and B and T cells do so.

To our knowledge, our study is the first proof of principle study that analyzes the labeling of HMPA polymers with scandium-44 and its in vivo distribution. Besides high in vitro stabilities, the good in vivo stability enables imaging of biological processes for up to 24 h. Further studies will focus on the comparison of different molecular weight polymers for enhanced EPR-mediated enrichment into tumors as well as receptor-mediated targeting to tumors as it was shown for [^44^Sc]Sc-DOTA-NAPamide for melanoma imaging [51].

## Conclusions

This study confirms the principle applicability of HPMA conjugates for labeling with different metallic radionuclides with established radiolabeling chemistry. Introducing as less than only 1.6% of DOTA chelator using a short linker chain, which facilitates the synthesis and efficiency of the polymer conjugates, allows introduction of both diagnostic and therapeutic radiometals. The introduction of [^44^Sc]Sc-DOTA as well as lutetium-177 into HPMA polymers allows for longer observation times than for example ^18^F-labeling being a good prerequisite for tumor imaging and therapy in the future.

## Additional file

Additional file 1: Supporting information. (ZIP 77 kb)

## Abbreviations

DOTA: 1,4,7,10-Tetraazacyclododecane-1,4,7,10-tetraacetic acid
DTPA: Diethylenetriaminepentaacetic acid
EDTA: Ethylenediaminetetraacetic acid
EPR: Enhanced permeability and retention
GPC: Gel permeation chromatography
HPMA: *N*-(2-Hydroxypropyl)methacrylamide
HSA: Human serum albumin
NaCl: Sodium chloride
NMR: Nuclear magnetic resonance
PDI: Polydispersity index
PET: Positron emission tomography
THF: Tetrahydrofuran
TLC: Thin-layer chromatography
