# Supplementary material for: Labeling of DOTA-conjugated HPMA-based polymers with trivalent metallic radionuclides for molecular imaging
Source: EJNMMI Res. 2018 Feb 27;8:16. doi: 10.1186/s13550-018-0372-x (PMC5829281; doi:10.1186/s13550-018-0372-x)
Supplement: Supplementary file 1 — Supporting information. (ZIP 77 kb) [file 13550_2018_372_MOESM1_ESM.zip › Supporting Information_revision.docx]

**Supporting Information**

**Contents**

1 Experimental section

I. Synthesis of Benzyl-phenyl-carbonate

II. Synthesis of mono-Cbz-protection of unprotected alkanediamines

III. Synthesis of mono-Cbz-protection of unprotected alkoxydiamines

IV. Coupling of mono-Cbz-protected spacers to DOTA-(tBu)_3_

V. Hydrogenation

VI. Synthesis of 4-cyano-4-((thiobenzoyl)sulfanyl)pentanoic acid (CTP)

VII. Synthesis of Pentafluorophenyl methacrylate (PFPMA)

VIII. Synthesis of reactive ester homopolymers (macro-CTA) (24)

IX. Removal of dithioester end groups

X. Synthesis of DOTA derivatives

XI. Deprotection of homopolymers

XII. Radiolabelling of polymers with Ga-68

XIII. *In vitro* stability

2 References

3 Supplemental Figures and Tables

**1 Experimental**

*I. Synthesis of Benzyl-phenyl-carbonate*

Benzyl alcohol (0.64 mol, 69.21 g) and pyridine (0.79 mol, 64 mL) were mixed with 175 mL CH_2_Cl_2_. Within 1 h phenylchloroformate (0.64 mol, 100.21 g) was added drop wise. The reaction mixture was stirred for 3 h and then added to 250 mL H_2_O. The organic phase was washed 2 x with 250 mL 2 M H_2_SO_4_, dried and concentrated under vacuum. Excess of benzyl alcohol was removed under vacuum and the residue dried under vacuum. The method gave 142.85 g (0.63 mol; 98 % yield) product as white solid. ^1^H NMR (300 MHz, CDCl_3_) δ (ppm) = 5.28 (s, 2H), 7.18 – 7.28 (m, 3H), 7.36 – 7.46 (m, 7H). MS (ESI) m/z (% rel Int) = 229.30 [M+H]^+^, 251.35 [M+Na]^+^. Calculated: 229.09 [M+H]^+^, 251.07 [M+Na]^+^

*II. Synthesis of mono-Cbz-protection of unprotected alkanediamines*

Benzyl-phenyl-carbonate (5 mmol, 1.141 g) in 5 mL EtOH was added drop wise over a period of 1 h to a solution of α,ω-diamine (5 mmol) in 25 mL abs. EtOH at 0 °C. The reaction mixture was stirred at room temperature for 2 days, concentrated under vacuum and the residue added to 25 mL H_2_O. The pH was adjusted to 3 with 4 N HCl and the aqueous phase extracted 3 x with 50 mL CH_2_Cl_2_. After adjusting the pH with 2 N NaOH to 11 N the aqueous phase was extracted 3x with 80 mL CH_2_Cl_2_. The organic phase was dried with MgSO_4_ and evaporated under reduced pressure. The residue was then dried under vacuum to yield the product as white solid.

- Benzyl-2-aminoethylcarbamate (7)

(85 % yield). ^1^H NMR (300 MHz, CDCl_3_) δ (ppm) =1.43 (s, 2H), 2.75 – 2.79 (t, 2H), 3.17 – 3.21 (m, 2H), 5.08 (s, 2H), 5.44 (bs, 1H), 7.28 – 7.34 (m, 5H). MS (ESI) m/z (% rel Int) = 195.11 [M+H]^+^, 217.10 [M+Na]^+^. Calculated: 195.11 [M+H]^+^, 217.09 [M+Na]^+^

- Benzyl-6-aminohexylcarbamate (8)

(67 % yield). ^1^H NMR (300 MHz, CDCl_3_) δ (ppm) =1.25 (s, 2H), 1.32 – 1.50 (m, 8H), 2.65 – 2.69 (t, 2H), 3.15 – 3.20 (m, 2H), 5.09 (s, 2H), 5.44 (bs, 1H), 7.32 – 7.37 (m, 5H). MS (ESI) m/z (% rel Int) = 251.17 [M+H]^+^, 274:20 [M+Na]^+^. Calculated: 251.18 [M+H]^+^, 273.16 [M+Na]^+^

- Benzyl-12-aminododecylcarbamate (9)

(50 % yield). ^1^H NMR (300 MHz, CDCl_3_) δ (ppm) =1.25 (s, 16H), 1.40 – 1.50 (m, 4H), 2.67 – 2.72 (t, 2H), 3.15 – 3.22 (m, 2H), 5.09 (s, 2H), 5.44 (bs, 1H), 7.33 – 7.37 (m, 5H). MS (ESI) m/z (% rel Int) = 335.26 [M+H]^+^, 357.26 [M+Na]^+^. Calculated: 335.27 [M+H]^+^, 357.25 [M+Na]^+^

*III. Synthesis of mono-Cbz-protection of unprotected alkoxydiamines*

Alkoxydimamine (20 mmol) and Na_2_CO_3_ (2 g) were mixed in 50 mL CHCl_3_ and cooled to 0°C. To this solution Cbz-Cl (10 mmol, 1.612 g) was added drop wise within 2 h. The mixture was stirred 2 h at 0°C, then 1 day at RT. After concentration the residue was added to 25 mL H_2_O. pH was adjusted to 3 with 4 N HCl and then the mixture was extracted 3x with 50 mL CH_2_Cl_2_. After adjusting the pH with 2 N NaOH to 11 the aqueous phase was extracted 3x with 80 mL CH_2_Cl_2_. The organic phase was dried with Na_2_SO_4_ and concentrated under reduced pressure. The residue was dried under vacuum to yield the product as white solid.

- Benzyl-2-(2-(2-aminoethoxy)ethoxy)ethylcarbamate (10)

(47 % yield). ^1^H NMR (300 MHz, CDCl_3_) δ (ppm) =1.53 (s, 2H), 2.78 – 2.81 (t, 2H), 3.32 – 3.37 (m, 2H), 3.43 – 3.49 (m, 6H), 3.50 – 3.59 (m, 6H), 5.06 (s, 2H), 7.28 – 7.32 (m, 5H). MS (ESI) m/z (% rel Int) = 283.12 [M+H]+, 305.14 [M+Na]^+^. Calculated: 283.17 [M+H]^+^, 305.15 [M+Na]^+^

- Benzyl-3-(4-(3-aminopropoxy)butoxy)propylcarbamate (11)

(39 % yield). ^1^H NMR (300 MHz, CDCl_3_) δ (ppm) =1.56 – 1.67 (m, 8H), 2.74 – 2.76 (m, 2H), 3.22 – 3.29 (m, 2H), 3.36 – 3.45 (m, 10H), 5.05 (s, 2H), 7.27 – 7.32 (m, 5H). MS (ESI) m/z (% rel Int) = 339.20 [M+H]^+^, 361.24 [M+Na]^+^. Calculated: 339.23 [M+H]^+^, 361.21 [M+Na]^+^

*IV. Coupling of mono-Cbz-protected spacers to DOTA-(tBu)_3_*

DOTA-(tBu)_3_ (0.035 mmol; 20 mg), mono-Cbz-protected amine (0.052 mmol) and TMP (0.08 mmol; 9 mg) were dissolved in 5 mL NMP and cooled to 0°C. COMU (0.055 mmol; 23.5 mg) was dissolved in 2 mL NMP, added drop wise to the mixture and stirred 15 min at 0°C and 2 h at RT. The mixture was then diluted with 20 mL EtOAc and extracted 3x with 20 mL 0.1 N HCl, NaHCO_3_ and Brine. The organic phase was dried and concentrated under reduced pressure. The product was achieved as colourless oil.

-^t^Butyl-2,2´,2´´-(10-(2-(2-(benzoyloxycarbonylamino)ethylamino)-2-oxoethyl)-1,4,7,10-tetraazacyclodo-decane-1,4,7-triyl)triacetate (12)

(75% yield). ^1^H NMR (300 MHz, CDCl_3_) δ (ppm) =1.45 (s, 27H), 1.63 (s, 8H), 2.50 – 3.50 (bm, 20H), 5.06 (s, 2H), 7.31 – 7.35 (m, 5H). MS (ESI) m/z (% rel Int) = 749.48 [M+H]+, 771.36 [M+Na]^+^. Calculated: 749.48 [M+H]^+^, 771.46 [M+Na]^+^

-^t^Butyl-2,2´,2´´-(10-(2-(6-(benzoyloxycarbonylamino)hexylamino)-2-oxoethyl)-1,4,7,10-tetraazacyclodo-decane-1,4,7-triyl)triacetate (13)

(72% yield). ^1^H NMR (300 MHz, CDCl_3_) δ (ppm) =1.15 – 1.30 (m, 10H), 1.33 - 153 (s, 27H), 1.98 (s, 2H) 2.32 – 3.24 (bm, 24H), 5.07 (s, 2H), 7.26 – 7.35 (m, 5H). MS (ESI) m/z (% rel Int) = 805.58 [M+H]+, 827.44 [M+Na]^+^. Calculated: 805.54 [M+H]^+^, 827.53 [M+Na]^+^

-^t^Butyl-2,2´,2´´-(10-(2-(12-(benzoyloxycarbonylamino)dodecylamino)-2-oxoethyl)-1,4,7,10-tetraazacyclo-dodecane-1,4,7-triyl)triacetate (14)

(66% yield). ^1^H NMR (300 MHz, CDCl_3_)) δ (ppm) =1.24 – 1.35 (m, 37H), 2.00 – 2.99 (s, 16H) 3.15 – 3.73 (bm, 10H), 5.05 (s, 2H), 7.28 – 7.39 (m, 5H). MS (ESI) m/z (% rel Int) = 889.64 [M+H]^+^, 911.59 [M+Na]^+^. Calculated: 889.64 [M+H]^+^, 911.62 [M+Na]^+^

-^t^Butyl-2,2´,2´´-(10-(3,14-dioxo-1-phenyl-2,8,10-trioxa-4,13-diazapentadecan-15-yl)-1,4,7,10-tetraaza-cyclododecane-1,4,7-triyl)triacetate (15)

(70% yield). ^1^H NMR (300 MHz, CDCl_3_) δ (ppm) =1.24 – 1.35 (m, 37H), 2.00 – 2.99 (s, 16H) 3.15 – 3.73 (bm, 10H), 5.05 (s, 2H), 7.28 – 7.39 (m, 5H). MS (ESI) m/z (% rel Int) = 837.58 [M+H]^+^, 859.50 [M+Na]^+^. Calculated: 837.53 [M+H]^+^, 859.52 [M+Na]^+^

-^t^Butyl-2,2´,2´´-(10-(3,18-dioxo-1-phenyl-2,8,13-trioxa-4,17-diazanondecan-19-yl)-1,4,7,10-tetraazacyclo-dodecane-1,4,7-triyl)triacetate (16)

(79% yield). ^1^H NMR (300 MHz, CDCl_3_) δ (ppm) =1.24 – 1.30 (m, 10H), 1.40 – 1.49 (s, 27H), 1.52 – 1.61 (m, 4H), 1.66 – 1.79 (m, 4H), 2.20 – 2.99 (s, 16H) 3.15 – 3.73 (bm, 20H), 5.05 (s, 2H), 7.28 – 7.39 (m, 5H). MS (ESI) m/z (% rel Int) = 893.52 [M+H]+, 915.55 [M+Na]^+^. Calculated: 893.60 [M+H]^+^, 915.58 [M+Na]^+^

*V. Hydrogenation*

Pd/C (30 mg), 0.06 mmol of the compounds (12-16) were mixed in 5 mL THF:MeOH (1:1) and stirred for 4 h under H_2_-atmosphere at RT. The mixture was passed over celite, concentrated and dried under vacuum. The product was achieved as colourless oil.

-^t^Butyl-2,2´,2´´-(10-(2-(2-ethylamino)-2-oxoethyl)-1,4,7,10-tetraazacyclododecane-1,4,7-triyl)triacetate (17)

(96% yield). ^1^H NMR (300 MHz, CDCl_3_) δ (ppm) =1.45 (s, 27H), 1.63 (s, 8H), 2.50 – 3.50 (bm, 20H). MS (ESI) m/z (% rel Int) = 637.43 [M+Na]^+^. Calculated: 637.43 [M+Na]^+^

-^t^Butyl-2,2´,2´´-(10-(2-(6-aminohexylamino)-2-oxoethyl)-1,4,7,10-tetraazacyclododecane-1,4,7-triyl)-triacetate (18)

(97 % yield). ^1^H NMR (300 MHz, CDCl_3_) δ (ppm) =1.15 – 1.30 (m, 10H), 1.33 - 153 (s, 27H), 1.98 (s, 2H) 2.32 – 3.24 (bm, 24H). MS (ESI) m/z (% rel Int) = 693.37 [M+Na]^+^. Calculated: 693.45 [M+Na]^+^

-^t^Butyl-2,2´,2´´-(10-(2-(12-aminododecylamino)-2-oxoethyl)-1,4,7,10-tetraazacyclododecane-1,4,7-triyl)-triacetate (19)

(93% yield). ^1^H NMR (300 MHz, CDCl_3_) δ (ppm) =1.24 – 1.35 (m, 37H), 2.00 – 2.99 (s, 16H) 3.15 – 3.73 (bm, 10H). MS (ESI) m/z (% rel Int) = 777.57 [M+Na]^+^. Calculated: 777.58 [M+Na]^+^

-^t^Butyl-2,2´,2´´-(10-(2-(2-(2-(2-aminoethoxy)ethoxy)ethylamino)-2-oxoethyl)-1,4,7,10-tetraazacyclodo-decane-1,4,7-triyl)triacetate (20)

(96% yield). ^1^H NMR (300 MHz, CDCl_3_) δ (ppm) =1.24 – 1.35 (m, 37H), 2.00 – 2.99 (s, 16H) 3.15 – 3.73 (bm, 10H). MS (ESI) m/z (% rel Int) = 725.50 [M+Na]^+^. Calculated: 725.48 [M+Na]^+^

-^t^Butyl-2,2´,2´´-(10-(2-(3-(4-(3-aminopropoxy)butoxy)propylamino)-2-oxoethyl)1,4,7,10-tetraazacyclo-dodecane-1,4,7-triyl)triacetate (21)

(94% yield). ^1^H NMR (300 MHz, CDCl_3_) δ (ppm) =1.24 – 1.30 (m, 10H), 1.40 – 1.49 (s, 27H), 1.52 – 1.61 (m, 4H), 1.66 – 1.79 (m, 4H), 2.20 – 2.99 (s, 16H) 3.15 – 3.73 (bm, 20H). MS (ESI) m/z (% rel Int) = 781.52 [M+Na]^+^. Calculated: 781.54 [M+Na]^+^

For compounds 17-21 no values could be detected for [M+H]^+^.

*VI Synthesis of 4-Cyano-4-((thiobenzoyl)sulfanyl)pentanoic acid (CTP) (22)*

4-Cyano-4-((thiobenzoyl)sulfanyl)pentanoic acid was used as chain transfer agent (CTA) and synthesized according to the literature (1).

*VII. Synthesis of p*entafluorophenyl methacrylate (PFPMA) (23)

Pentafluorophenyl methacrylate was prepared according to reference (2).

*VIII.* Synthesis of reactive ester homopolymers (macro-CTA) (24)

Reversible addition-fragmentation chain transfer (RAFT) polymerization of pentafluorophenyl methacrylate with 4-cyano-4-((thiobenzoyl)sulfanyl)pentanoic acid was carried out in a schlenk tube (3, 4). For this purpose, 4 g of PFPMA were dissolved in 5 mL of absolute dioxane, furthermore CTP and AIBN were added. The molar ratio of CTP/AIBN was chosen 1:8. After three freeze-vacuum-thaw cycles, the mixture was immersed in an oil bath at 65 °C and stirred overnight. Afterwards, the polymeric solution was precipitated three times in hexane, centrifuged and dried under vacuum at 40 °C overnight. A slightly pink powder was obtained. (52 % yield). ^1^H-NMR (300 MHz, CDCl_3_) δ (ppm): 1.20-1.75 (br), 2.00-2.75 (br s). ^19^F-NMR (400 MHz, CDCl_3_) δ (ppm): -162.03 (br), -156.92 (br), -152 to -150 (br)

*IX. Removal of dithioester end groups*

The dithiobenzoate end group was removed using the protocol reported by Perrier et al., 2005 (5). Therefore a 25-fold molar excess of AIBN was added to the polymer dissolved in dioxane. After 4 h of heating the solution in an oil bath at 70 °C, the polymer was precipitated twice in hexane and collected by centrifugation. The polymer was dried under vacuum overnight, a colourless powder was obtained. Yield: 75 %. Removal of the dithioester end group could be proven by UV-Vis spectroscopy.

*X. Synthesis of DOTA derivatives*

For radioactive labeling of homopolymers the protocol was applied as follows. 180 mg of poly(PFPMA) homopolymer was dissolved in 2 mL of absolute dioxane. 0.11 mmol of DOTA-spacer (17-21) and 11 mg of triethylamine were diluted in abs. DMSO and added to the vessel. After stirring for 36 h at 45 °C, 45 mg of 2-hydroxypropylamine and 61 mg of Et_3_N were added and the solution further stirred for 48 h. For final removal of reactive ester side groups 45 mg of 2-hydroxypropylamine were additionally added the next morning. The solution was finally prepared in a DMSO/water mixture for dialysis. After lyophilisation a white, crystalline powder could be obtained. (51 %-55 % yield). ^1^H-NMR (400 MHz, d. DMSO) δ (ppm): 0.60-1.40 (br), 1.35-1.45 (s), 1.45-2.20 (br), 2.75-3.10 (br), 3.50-3.80 (br), 4.60-4.80 (br)

*XI. Deprotection of homopolymers*

The polymer was dissolved in acetonitrile and TFA was added (1:1). This solution was stirred 4 h at RT. For purification dialysis over 2 days was used. The product was obtained as white powder. Yield: 89-92 %; (Exception: It was not possible to recover the purified conjugate (32) from the dialysis flexible tube) ^1^H-NMR (400 MHz, d. DMSO) δ (ppm): 0.60-1.40 (br), 1.45-2.20 (br), 2.75-3.10 (br), 3.50-3.80 (br), 4.60-4.80 (br)

*XII. Radiolabelling of polymers with gallium-68*

Acetone post-processed eluate 400 µL N2 (97.56 % acetone/0.05 N HCl) containing 40-60 MBq of gallium-68, was added to 3 mL 1 M NaAcetate containing the polymer and the reaction mixture was heated up to 95°C. Additionally labeling with ethanol post-processed eluate was performed for conjugates (33-35). 1 mL N5 (90 % ethanol/0.9 N HCl) was added to 3 mL 1 M NaAcetate buffer containing polymer.

XIII. *In vitro stability*

Stability studies were performed with ^68^Ga-labeled polymers of radiochemical purity > 98 %. Stability studies in 0.9 % NaCl and human serum were performed by addition 50 μL of purified product to 500 μL of pre-warmed NaCl or human serum. Solutions were incubated for 2 h at 37 °C. Stability of ^68^Ga-labeled product was also monitored in solutions containing different metal cations (Fe^3+^, Ca^2+^, Mg^2+^) at concentration levels of 10^-2^ M each. 50 μL purified product were added to 500 μL of an aqueous solution containing one of the metal cations. Solutions were incubated for 2 h at 37 °C. Studies with ETDA or DTPA solutions were also performed to check the stability of labeled product in presence of competing chelating ligands. The aliquot of purified product were added to DTPA or EDTA solutions in 0.9 % NaCl with a final molar ratio of 100:1. The final volume of the solution was 550 μL. Solutions were incubated for 2 h at 37 °C. From all studied solutions aliquots were taken after 1, 10, 25, 40, 60, 80 and 120 min and analysed by TLC.

1. **REFERENCES**

1. Moad G, Rizzardo E, Thang SH. Living Radical Polymerization by the RAFT Process. Australian Journal of Chemistry. 2005;58(6):379-410.

2. Eberhardt M, Mruk R, Zentel R, Théato P. Synthesis of pentafluorophenyl(meth)acrylate polymers: New precursor polymers for the synthesis of multifunctional materials. European Polymer Journal. 2005;41(7):1569-75.

3. Theato P. Synthesis of well-defined polymeric activated esters. Journal of Polymer Science Part A: Polymer Chemistry. 2008;46(20):6677-87.

4. Barz M, Luxenhofer R, Zentel R, Kabanov AV. The uptake of N-(2-hydroxypropyl)-methacrylamide based homo, random and block copolymers by human multi-drug resistant breast adenocarcinoma cells. Biomaterials. 2009;30(29):5682-90.

5. Perrier S, Takolpuckdee P, Mars CA. Reversible Addition-Fragmentation Chain Transfer Polymerization: End Group Modification for Functionalized Polymers and Chain Transfer Agent Recovery 2005 [15.02.2005:[2033-6].

6. Roesch F, Perez-Malo Cruz M. Improved efficacy of synthesis of 68Ga radiopharmaceuticals in mixtures of aqueous solution and non-aqueous solvents. Journal of Nuclear Medicine. 2013;54(supplement 2):163.

7. Eppard E, Wuttke M, Nicodemus PL, Rosch F. Ethanol-Based Post-processing of Generator-Derived (6)(8)Ga Toward Kit-Type Preparation of (6)(8)Ga-Radiopharmaceuticals. J Nucl Med. 2014;55(6):1023-8.

1. **SUPPLEMENTAL FIGURES and Tables**

Figure S1: Comparison of labeling yields of **33** using EtOH and acetone post-processed gallium-68 (95° C; n = 3).

Table S1: Biodistribution data of the 44Sc-31c polymer in B16-bearing mice 2, 4 and 24 hours after i.v. injection (corresponding to Figure 10). Data is represented in % ID/g tissue (mean±SD). N= 5 mice per group.

|  | **^44^Sc-31c** | | |  |
| --- | --- | --- | --- | --- |
| **organ** | **2 h** | **4 h** | **24 h** | |
| lung | 5.76±2.42 | 4.37±0.59 | 6.79±0.66 | |
| blood | 15.48±1.79 | 16.57±3.66 | 5.52±1.54 | |
| liver | 3.83±0.51 | 3.24±0.6 | 5.39±0.47 | |
| spleen | 3.21±0.52 | 2.34±0.42 | 9.36±0.89 | |
| Kidney r | 30.51±7.32 | 40.17±7.68 | 26.34±7.35 | |
| Kidney l | 35.39±6.13 | 40.68±6.8 | 36.56±9.55 | |
| muscle | 0.75±0.09 | 0.79±0.16 | 5.59±0.91 | |
| heart | 3.81±0.44 | 3.12±0.49 | 4.94±0.43 | |
| urine | 54.57±9.22 | 73.58±11.5 | 32.41±10.2 | |
| intestine | 2.92±0.73 | 2.12±0.59 | 5.54±1.19 | |
| testicles | 1.98±0.41 | 2.04±0.5 | 5.51±0.95 | |
| tumor | 4.44±0.65 | 5.07±1.21 | 8.18±2.58 | |
