# Supplementary figures and images for: Labeling of DOTA-conjugated HPMA-based polymers with trivalent metallic radionuclides for molecular imaging
Source: EJNMMI Res. 2018 Feb 27;8:16. doi: 10.1186/s13550-018-0372-x (PMC5829281; doi:10.1186/s13550-018-0372-x)

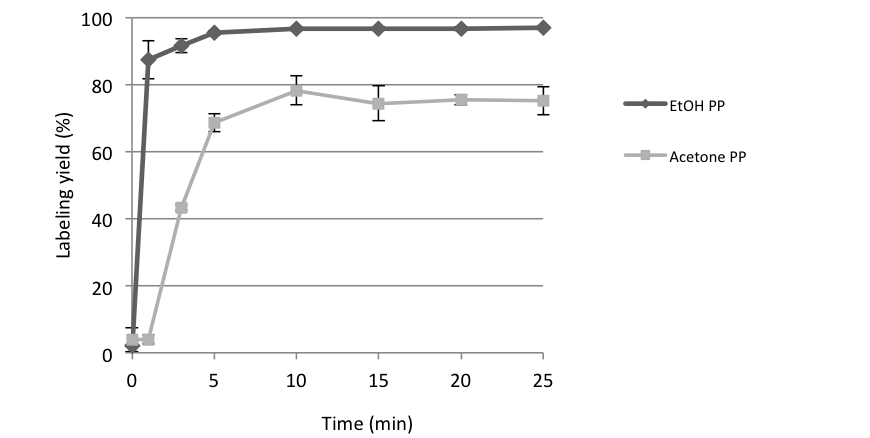

Supplement: Supplementary file 1 — Supporting information. (ZIP 77 kb) [file 13550_2018_372_MOESM1_ESM.zip › figure S1.png]
